# Rebound of platelet count in a patient with type 2 calreticulin-mutant essential thrombocythemia in the postpartum period: A case report

**DOI:** 10.1097/MD.0000000000027725

**Published:** 2021-11-05

**Authors:** Abdulrahman F. Al-Mashdali, Mohamed A. Yassin

**Affiliations:** a Department of Internal Medicine, Hamad Medical Corporation, Doha, Qatar; b National Center for Cancer Care and Research, Department of Oncology, Hematology and BMT Section, Hamad Medical Corporation, Doha, Qatar.

**Keywords:** calreticulin, case report, essential thrombocythemia, Pegylated Interferon alfa, postpartum period, rebound of platelet count

## Abstract

**Introduction:**

Essential thrombocythemia (ET) is an uncommon myeloproliferative neoplasm. It is more common in females; 20% of them are below 40 years old. The optimal management of ET during pregnancy and postpartum periods is still not well established.

**Patient concern:**

We report a case of a young lady with type 2 calreticulin-mutant ET who developed a marked rebound in her platelet count (reaching 2030 × 10^3^/μL) 2 weeks after premature delivery of her baby (24^th^ week of gestation). She was on Pegylated Interferon alfa 2-a during pregnancy (her platelet was around 500 × 10^3^/μL during the second trimester), but she had stopped it on her own from the 20^th^ week of gestation.

**Diagnosis:**

Postpartum rebound of platelet count due to medication non-compliance.

**Intervention and outcome:**

We resumed her regular Pegylated Interferon, and subsequently, her platelet count reduced dramatically within 4 weeks to an acceptable level (684 × 10^3^ /μL).

**Conclusion:**

The guideline is still not well-established regarding the optimal approach for postpartum rebound of platelet count in patients with ET. It is still unclear if the platelet count will fall spontaneously without intervention after the rebound phase. Further research is required to establish the optimal management of ET during the postpartum phase. This case emphasizes the importance of platelet count follow-up during the postpartum period and outlines our management approach in such cases.

## 1 Introduction

Myeloproliferative neoplasms (MPNs), formerly named myeloproliferative disorders, are a group of diseases characterized by the excessive clonal production of red blood cells, platelets, or white blood cells, mainly in the bone marrow and occasionally in the liver and generally classified as Philadelphia positive MPN which is represented by chronic myeloid leukemia^[1]^; and Philadelphia negative MPNs including, essential thrombocythemia (ET), polycythemia vera, and idiopathic myelofibrosis.^[2]^ ET is an uncommon MPN characterized by the overproduction of platelets by bone marrow megakaryocytes.^[3]^ Eventually, it may progress to myelofibrosis or acute myeloid leukemia.^[4]^ ET is usually sporadic and rarely familial.^[5]^ Based on the latest World Health Organization criteria for MPNs, ET is considered as a diagnosis of exclusion.^[6]^

ET is more prevalent in females, and approximately 80% of them are above 40 years old. Nonetheless, ET still occurs in women within reproductive age.^[7]^ ET increases the risk of thrombotic and bleeding events.^[8]^ Pregnancy is one of the hypercoagulable states,^[9]^ and pregnant women with ET are at high risk for thrombosis and its complications on the mother and fetus.^[10]^ Thus, several guidelines and experts recommend ET management with interferon (IFN) therapy during pregnancy, especially in patients at high risk for pregnancy loss.^[11]^

Interestingly, many patients with ET may develop a rebound in platelet count during the postpartum course, which increases the risk of thrombosis during this prothrombotic period.^[11]^ Herein, we present a case of type 2 calreticulin-mutant ET, who stopped her regular Pegylated Interferon Alfa-2a dose during pregnancy on her own and developed a marked rebound in her platelet counts during the postpartum period.

## 2 Case presentation

A 30-year-old female was referred to our hematology clinic due to the persistent elevation in her platelet count. The patient also reported fatiguability and generalized body pain for few months. Her past medical and family history was unremarkable. Complete blood count revealed a platelet count of 2300 × 10^3^/μL. Other relevant laboratory results on presentation are shown below in Table 1. Physical examination was normal (no organomegaly or lymphadenopathy). MPN, specifically ET, was suspected, and the workup was initiated to confirm that. The bone marrow biopsy result was consistent with ET. The test for driver gene mutation was positive for a type 2, 5 bp insertion mutation within exon 9 of the calreticulin (CALR) gene. Janus kinase 2 (JAK2V617F) and *BCR-ABL1* gene fusion mutations were negative.

**Table 1:** Laboratory findings on the first presentation.

	Value	Normal range
*WBC*	*15.20 × 10* ^*3*^ */μL*	4.00–10.00
RBC	4.0 × 10^3^/μL	3.8–4.8
*Hgb*	*12.0* *gm/dL*	12.0–15.0
Hct	36.4%	36.0–46.0
MCV	91.0 fL	83.0–101.0
*Platelet*	*2030 × 10* ^*3*^ */μL*	150–400
ANC	11.2 × 10^3^/μL	2.0–7.0
Lymphocyte	2.8 × 10^3^/μL	1.0–3.0
Monocyte	1.0 × 10^3^/μL	0.2–1.0
Eosinophil	0.2 × 10^3^/μL	0.0–0.5
Basophil	0.10 × 10^3^/μL	0.02–0.10
*vWF Ag*	*66.6%*	
vWF activity	35.2 s	
*C-reactive protein*	*<5* *mg/L*	
Creatinine	50 μmol/L	44–80
Sodium	137 mmol/L	136–145
Potassium	6.1 mmol/L	3.5–5.1
Adjusted calcium	2.56 mmol/L	2.20–2.55
Albumin	34.0 gm/L	35.0–52.0
ALT	9 U/L	0–33
AST	15 U/L	0–32

ALT = alanine aminotransferase, ANC = absolute neutrophil count, AST = aspartate aminotransferase, Hct = hematocrit, Hgb = hemoglobin, MCV = mean corpuscular volume, RBC = red blood cell, vWF = von Willebrand factor (VWF), WBC = white blood cell.

**Figure 1:**
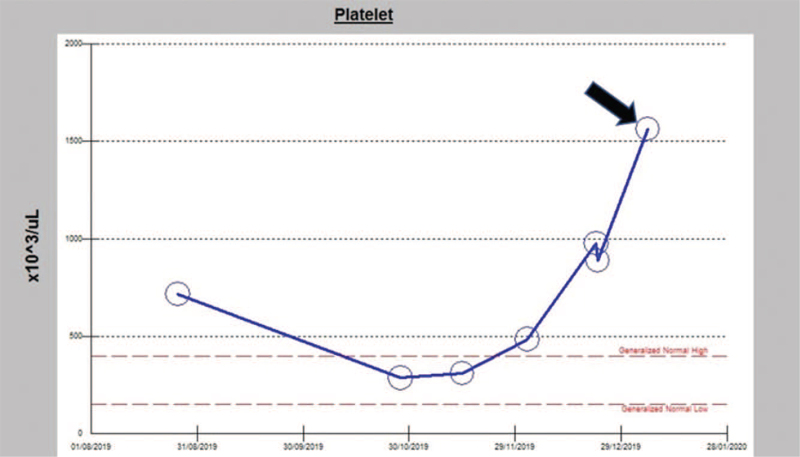
Trend in the patient platelet counts during the late pregnancy and postpartum period (The black arrow shows the rebound in the platelet count during the postpartum period).

The ET diagnosis was confirmed, and the patient started on Pegylated Interferon Alfa-2a at a dose of 135 μg weekly. Her platelet count was controlled for several months after the initiation of therapy. However, during her second child's pregnancy, the patient stopped taking her weekly dose of Peginterferon Alfa-2a in the 20^th^ week of gestation without seeking medical advice. After that, she delivered her baby prematurely at the 24^th^ week of gestation through vaginal delivery. Her baby was intubated and admitted to the neonatal intensive care unit. Two weeks after delivery, during the postnatal follow, the patient was found to have a platelet count of 2030 × 10^3^/μL. However, the period after delivery was uneventful. Figure 1 shows the platelet counts trend during her pregnancy and the early postpartum period. We resumed Peginterferon Alfa-2a at a dose of 135 μg (weekly dose). After 4 weeks of follow-up, her platelet count decreased to 684 × 10^3^/μL.

Recently, the patient presented to the emergency department with a platelet count of 2538 × 10^3^/μL. No complications were identified. Again, the reason for the rebound in her platelet count was non-compliant with Peginterferon Alfa-2a. We added a 1-month course of hydroxyurea 100 mg twice a day to suppress the bone marrow production of platelets. Her latest platelet is currently controlled at 778 × 10^3^/μL on Peginterferon alfa-2a 135 mcg weekly with regular follow-up at the outpatient clinic until the moment.

## 3 Discussion

ET remains the most encountered MPN in young women within reproductive age.^[12]^ As mentioned in the introduction, ET patients who get pregnant are at higher risk for thrombotic complications. It was found that the rate of first-trimester spontaneous abortions and premature deliveries in ET patients were higher than that in healthy individuals.^[11]^ Additionally, pregnancy complications, like intrauterine growth restriction and placental abruption, are common in pregnant ladies with ET.^[13]^ Treatment of ET has been shown to improve the maternal and fetal outcomes during pregnancy. IFN-alpha therapy is considered the drug of choice for ET treatment during pregnancy.^[14]^ Our patient had stopped her Peginterferon alfa by the end of the first half of pregnancy. Unfortunately, her platelet counts increased substantially, and she delivered her baby prematurely at the 24^th^ week of gestation, supporting the relationship between uncontrolled platelet count in ET with worse pregnancy outcomes.

Janus kinase 2 (JAK2V617F), myeloproliferative leukemia (MPL), and CALR gene mutations have been detected in the majority of patients with ET. JAK2(V617F) mutation and MPL gene mutation have been reported in up to 60% and 5% of ET cases, respectively. Most of the ET cases that were negative for JAK2 and MPL genetic mutation were positive for CALR mutation.^[15]^ However, approximately 10% to 15% of cases are negative for all 3 mutated genes “triple-negative”. CALR is a calcium-binding protein located abundantly in the endoplasmic reticulum and involved in variable biological functions. CALR regulates the folding process of newly synthesized glycoprotein and contributes to calcium hemostasis.^[16,17]^ It was recently found that CALR mutation independently activates the thrombopoietin receptor, promoting megakaryocyte proliferation and, subsequently, ET development. There are 2 main types of CALR gene mutation, A 52-bp deletion (type 1 mutation) and a 5-bp insertion (type 2 mutation). Type 1 mutation is related to a higher risk of transformation to myelofibrosis. Whereas, type 2 variant, which was detected in our patient, is strongly linked to ET with an indolent disease course, higher platelet counts, and lower risk of thrombosis.^[18]^

Typically, platelet counts decline during pregnancy and eventually return to pre-gestational level within the first 4 to 6 weeks after delivery. The mechanism for this phenomenon is unclear. However, this decline in platelet count might play a protective role against thrombosis during the pregnancy (hypercoagulable state).^[9]^ As mentioned above, many patients with ET develop a rebound in platelet count after delivery. Experts suggest that platelet count should be repeated after 6 weeks of delivery.^[14]^ Notably, the platelet count's rebound may occur as early as 2 weeks postpartum, like in our patient and other reported cases. The data are still scarce regarding the optimal management of platelet count rebound during the postpartum period. The cytoreductive medication was initiated in some cases during the postpartum period, whereas in other cases, conservative management was sufficient with a spontaneous decline of platelet count. Most experts agree that the management of ET during the postpartum period should be individualized according to the disease-related factors and patient risk of thrombosis.^[19]^ We managed our patient by resuming her usual dose of Peginterferon Alfa-2a, resulting in a dramatic response with more than 50% reduction in her platelet count within a short period.

## 4 Conclusion

Our patient developed a significant rebound in her platelet count during the early postpartum period. However, she did not develop any thrombotic or bleeding complications during that time. Evidence for management of platelet rebound after delivery is still scarce. It is still unclear if the platelet count will fall spontaneously without intervention after the rebound phase. Accordingly, the platelet count should be followed closely during this period, optimally every week. We highly suggest IFN therapy, considering its safety profile and efficacy to lower platelet count, to prevent thrombotic events during this prothrombotic period. Further research is required to establish the optimal management of ET during the postpartum phase.

## Author contributions

**Conceptualization:** Abdulrahman Fadhl Al-Mashdali, Mohamed A. Yassin.

**Data curation:** Abdulrahman Fadhl Al-Mashdali, Mohamed A. Yassin.

**Writing – original draft:** Abdulrahman Fadhl Al-Mashdali, Mohamed A. Yassin.

**Writing – review & editing:** Abdulrahman Fadhl Al-Mashdali, Mohamed A. Yassin.
